# Character Virtues: Toward a Functionalist Perspective on Character Virtue Science

**DOI:** 10.3390/bs15050638

**Published:** 2025-05-07

**Authors:** Navrose Bajwa, Vincent Ng

**Affiliations:** Department of Psychology, College of Liberal Arts and Social Sciences, University of Houston, Houston, TX 77204, USA

**Keywords:** virtue, positive psychology, measurement, assessment, functionalist, character types

## Abstract

Contemporary psychology often reduces virtue to stable traits or observable behaviors, overlooking the motivational core that has long been central to classical virtue ethics. However, focusing narrowly on behaviors without considering intent is insufficient for virtue assessment because similar behaviors can stem from vastly different intentions, and both the behavior and its intention is definitional to what behaviors are considered virtuous. We draw on Aristotle’s five character types—beastly, vicious, incontinent, continent, and virtuous—in this paper. In doing so, we ultimately argue that a functionalist approach to character research is not only a useful alternative to the trait approach, but necessary to more fully capture the character virtues construct. At the same time, we recognize an epistemic boundary: psychology can only observe manifestations and reports of virtue, never virtue itself. We therefore distinguish carefully between descriptive evidence and the normative judgments required to label any act ‘virtuous’.

## 1. Introduction

Despite its longstanding prominence in moral philosophy, the integrated relationship between action and moral intention is sometimes lost in contemporary attempts to measure virtue. Indeed, some have argued that many psychological theories and models of virtues overlook the motivational component (e.g., Besser, 2017), specifically the intentions behind the virtue-relevant behavior (e.g., Fowers et al., 2021). Modern psychological interpretations often reduce virtues to stable traits or observable behaviors but ignore the role of motivations in virtues assessment, thereby stripping it of part of its defining essence and ignoring the role of doing the appropriate action well. Reducing it to a fixed disposition ignores that virtues do not merely consist in behavior, but also of the agent’s ability to discern, desire, and effectively pursue what is good for the right reasons. It requires a deep, habituated alignment of intellect, moral purpose, and emotional maturity, where reasoning and feeling are harmonized in the pursuit of excellence. Recognizing this issue, many psychologists have sought to align their models more closely with Aristotelian principles (e.g., McManus et al., 2024; Ng & Tay, 2020; Ratchford et al., 2024). As these psychologists emphasize, the philosophical conceptualizations of virtue ethics are often “lost in translation”, leading to incomplete interpretations and assessments.

Psychological methods, by definition, generate descriptive data like self-reports, behavioral traces, informant ratings. However, virtue is at least partly a normative ideal. Calling an action “courageous”, or a pattern of actions “virtuous”, therefore requires a value-laden inference that no psychometric instrument can perform unaided (Fowers et al., 2021). As this paper focused on the intentions behind behavior (virtue motivation), we confined ourselves to the empirical question: How do individuals represent, pursue, and integrate moral ends? We acknowledge, however, that a full assessment of virtue also demands a second, normative question: Should those ends, or their integration be regarded as virtuous? Therefore, we treated the Aristotelian telos of eudaimonia and the pursuit of constitutive goods as the criterion against which measures were judged. From this stance, three forms of validity guided the empirical jury:

- Criterion validity—scores should predict flourishing (eudaimonic well-being), successful goal-pursuit, and observable virtue-relevant acts (e.g., generosity predicting helping) (Fowers, 2014).
- Convergent validity—scores should correlate with theoretically allied constructs (prosocial behavior, moral identity, individual differences in authentic functioning) and relate negatively to antithetical traits such as Machiavellianism (Fowers, 2014).
- Discriminant validity—scores should remain distinct from unrelated attributes (e.g., IQ, sex, race) because virtues are meant to transcend such categories (Fowers, 2008).

Choosing these validity standards is itself a normative act, but it lets empirical evidence shoulder much of the evaluative labor. As Fowers et al. (2021) note, virtue science must either disengage from evaluation or “bite the bullet” and declare its evaluative anchor. Therefore, any virtue-assessment project must make its position on this choice explicit.

While McManus et al. (2024) provided a comprehensive Aristotelian framework by measuring virtues across behavior, emotion, reasoning, and motivation, Ng and Tay (2020) demonstrated a golden-mean conceptualization emphasizing consistent, context-sensitive expressions of virtue, and Ratchford et al. (2024) explicitly defined virtues in Aristotelian terms. The present paper differs distinctly by placing an emphasis on the intent as well as appropriateness of the action, specifically, the unique interplay of reason and motivation in driving virtuous action. Moving beyond general motivational considerations, we argue that understanding an agent’s ends or purposes (why one is motivated) and how equipped they are in pursuing them is essential for accurately capturing virtue as Aristotle originally conceived it.

This paper argues for an expanded perspective: to fully capture virtue, we must consider not just how people behave, but *why* they behave that way. Organizing our discussion around Aristotle’s character types and their motivational continuum from beastly to vicious, incontinent, continent, and finally virtuous, we will show why motivation is important to understanding character. We then propose that a functionalist perspective is required to provide a theory-based approach to the assessment of virtues.

## 2. Character Types in Aristotelian Ethics

Aristotle identified five key character types: beastly, vicious, incontinent, continent, and virtuous. These categories capture both external actions and the internal motivations shaping them, highlighting why intent is integral to virtue. This helps us grapple with the idea that true virtue requires both the right action and the right intention.

**Beastly Character.** A beastly individual is entirely consumed by basic appetites, having lost the capacity for rational deliberation or moral self-regulation. Their actions are dictated by raw impulse, leading them to violate social and ethical norms without any sense of inner conflict. Behaviors such as stealing, lying, or even harming others may be undertaken solely to gratify an uncontrollable craving. In this state, reason and moral sensibility are effectively overrun by compulsive desires and inappropriate actions, resulting in a near-complete rejection of moral agency (Fowers, 2008).

**Vicious Character.** A vicious person consistently chooses morally wrong actions without inner conflict, believing such actions to be justified. They pursue harmful ends such as greed or exploitation. The vicious individual’s alignment of reason and desire is intact, but is geared toward destructive (e.g., epistemic malevolence; Baehr, 2020) or self-serving goals. In corporate settings, for example, a leader who systematically deceives stakeholders to maximize profit, experiences little or no internal conflict, and actively rationalizes their wrongdoing instead of struggling against it (Fowers, 2008).

**Incontinent Character.** An incontinent person knows what the right action is but repeatedly fails to follow through, overcome by conflicting desires. Despite recognizing the moral good, they often yield to impulses such as fear, selfishness, or laziness. They experience guilt, shame, or regret when they cannot uphold their better judgment. For instance, a manager who values honesty but regularly withholds crucial information due to fear of backlash exhibits incontinence, a troubling gap between moral knowledge and moral action. They feel guilty afterward, signifying that they know better yet cannot overcome their immediate reluctance or anxiety (Fowers, 2008).

**Continent Character.** A continent person also experiences inner conflict between moral duty and contrary desires. However, unlike the incontinent, the continent individual’s willpower triumphs: they do what is right despite not wanting to. By suppressing conflicting desires, they manage to act ethically. For example, a researcher who is in a foul mood or finds informed consent procedures inconvenient but forces themselves to follow the ethical protocols exemplifies continence. Although laudable, these actions are marked by effort and reluctance, indicating that the person has not fully internalized moral goodness (Fowers, 2008).

**Virtuous Character.** A virtuous person embodies Aristotle’s ideal: reason, desire, and emotion are in harmony, such that acting ethically feels natural and fulfilling. Motivated by phronesis (practical wisdom) and a commitment to noble ends, a virtuous individual does the right thing gladly. They do not merely perform correct actions; they act virtuously for its own sake. This internal alignment is the mark of eudaimonia, a life of flourishing wherein moral principles and personal inclinations coincide (Fowers, 2008; Aristotle, 1998).

Thus, Aristotle’s fivefold character typology systematically integrates external behaviors with internal psychological states, capturing the varied alignments between the motivation, reasoning, and emotional states that define moral character. Aristotle’s structured typology uniquely clarifies how distinct intentions and motivational dynamics systematically differentiate virtuous from non-virtuous actions. Consider, for example, the act of telling the truth under challenging circumstances:

1. A beastly character may impulsively lie without reflection, driven solely by immediate self-interest or fear, lacking the capacity to even recognize the moral significance of truth-telling.
2. A vicious character deliberately chooses deception, rationalizing it as justified or beneficial without experiencing internal conflict or remorse.
3. An incontinent character, although fully aware that honesty is morally right, succumbs to the temptation or fear of negative consequences and thus frequently lies, subsequently feeling guilt or regret.
4. A continent character similarly experiences intense temptation or fear but consistently manages, through significant internal effort and self-control, to uphold truthfulness and do the right thing despite inner reluctance.
5. A virtuous character, exemplifying moral excellence, naturally tells the truth even in difficult situations, not out of mere obligation or self-control, but because honesty genuinely aligns with their values and desires, making truthful action both effortless and fulfilling.

Aristotle’s typology thus helps systematically distinguish virtue from vice by clearly tracing how identical external behaviors (telling or not telling the truth) arise from critically different internal motivational structures and emotional states.

## 3. Why Motivation Matters: Critiques of Narrow Approaches

Below, we see how narrow focus on one dimension—traits, behaviors, or situations—struggle to distinguish among vicious, incontinent, continent, and virtuous individuals, primarily because it overlooks the role of motivation and internal conflict or alignment.

### 3.1. Trait-Based Approaches

Trait-based approaches in virtue psychology equate virtues with stable personality traits, emphasizing consistent behavioral patterns. These recognize that people have broad tendencies (e.g., “compassion trait level” to distinguish high from low compassion individuals) and offer a framework for measuring consistency or variability in behavior over time (Fleeson, 2001). However, these approaches are unable to differentiate internal motivations (Besser, 2017; Ahrens & Cloutier, 2019). Two individuals can display the same “helping” trait (e.g., consistently donating to charity) through relevant behavior, yet differ profoundly in reasons:

1. Virtuous vs. Continent: Both may donate regularly. However, the virtuous person gives out of genuine empathy, while the continent person might be motivated by a sense of moral duty but still struggles with reluctance or self-interest. Trait measures alone cannot capture this internal struggle.
2. Vicious or Incontinent: A vicious person might not donate at all or might only do so strategically to pursue an unethical goal, while an incontinent person wants to donate but often fails to do so due to weakness of will.

Trait-based approaches, which focus on relatively stable, genetically, and environmentally influenced between-person differences, do not fully account for the developmental aspect of moral character. Virtue involves a deliberate cultivation of moral character and the exercise of practical wisdom (phronesis); it is something a person actively develops rather than merely has. If we only look at traits as stable between-person dispositions, we miss the within-person dynamic processes by which individuals may intentionally refine their moral sensitivities, make wiser ethical judgments over time, and grow into virtuous persons (Besser, 2017; Ahrens & Cloutier, 2019; Korsgaard, 2008). Finally, motivation cannot be inferred from consistency alone. As Besser (2017) argues, acting “at the right time, toward the right people, for the right reason and in the right manner” is central to genuine virtue (Aristotle, 1962, sec. 1106b21-23; as cited in Besser, 2017). Simply knowing someone “does X consistently” reveals little about whether they are beastly, vicious, incontinent, continent, or virtuous.

In short, trait-based models struggle to distinguish, for example, a continent person’s reluctant but correct action from a virtuous person’s moral alignment, because both might manifest similar outward consistency.

### 3.2. Behavior-Based Approaches

Behavior-based models (e.g., the “summary view” linking virtue to visible actions) simplify moral evaluation to observable acts (Buss & Craik, 1983). While this might capture what someone does, it risks misidentifying character by neglecting why they act. Let us look at a vicious and incontinent person. Outwardly, both might commit the same harmful act of lying, cheating, or exploiting. However, the vicious person endorses such wrongdoing; they see it as justified or beneficial and feel no inner turmoil. In contrast, the incontinent person understands what is right, but repeatedly fails to follow through due to weak will or overpowering desires, experiencing remorse and frustration afterward. This distinction matters because a purely behavior-based view would lump both characters together as “doing bad things”, losing sight of key moral differences. The vicious person’s unconflicted alignment toward destructive ends contrasts sharply with the incontinent person’s regret and moral struggle. Consequently, Aristotelian ethics insists on examining not just the act itself, but also the actor’s motivations and the presence or absence of inner conflict. For Aristotle, virtue involves the person’s aim (telos), shaped by phronesis (practical wisdom) (Korsgaard, 2008). Behavior-based approaches can conflate “doing what looks good” with “doing what is truly good for the right reasons”.

Similarly, both continent and virtuous individuals may perform the same morally upright behavior. Behavior-based models would lump them together as “doing the right thing”. Aristotelian ethics, however, distinguishes them precisely by motivation and emotional alignment. The continent person overcomes reluctance, whereas the idealized virtuous person acts joyfully and wholeheartedly (Kristjánsson, 2008). Behavior certainly matters, but intentions and aims are also central to describing progress in character development from a neo-Aristotelian perspective.

### 3.3. Situation-Based Approaches

Situationist perspectives emphasize the power of external contexts to shape behavior, often citing classic studies (e.g., Milgram’s obedience experiments; Darley and Batson’s ‘Good Samaritan’ study) as evidence that behavior primarily depends on immediate situational factors rather than stable character traits or internal motivations. Situationists argue that seemingly stable traits, such as honesty or generosity, often vanish under subtle situational pressures, implying that consistent moral motives or intentions are of minimal relevance. From this viewpoint, what matters is not why someone behaves morally or immorally, but rather the external circumstances compelling these behaviors.

However, Aristotelian virtue ethics challenges this reductionist perspective by emphasizing that genuine moral character involves more than momentary reactions to external stimuli and specifically requires a cultivated and consistent integration of reason, emotion, and motivation. According to Aristotle, virtue is defined by integrated motive, reason, and consistent moral reflection. In other words, what matters is not merely whether a person momentarily acts honestly or not, but why they do so and how that pattern endures across time and situations.

Contemporary interactionist models effectively reconcile these divergent views. For instance, Fleeson’s (2001) density-distribution approach acknowledges substantial within-person behavioral variability, demonstrating that although people exhibit different trait-relevant behavior across contexts, they still maintain stable average trait dispositions over time (Jayawickreme et al., 2014). This suggests that individuals can flexibly respond to situational changes without entirely losing their core motivational structure. From this interactionist standpoint, even a virtuous person may occasionally experience pressures or temptations; however, due to their well-developed practical wisdom and habituated character, their virtuous motivations persist more consistently than those of less morally developed individuals.

Thus, while situation-based perspectives importantly highlight how external conditions shape behavior, they are incomplete without considering internal motivational and moral reasoning factors. Integrating Aristotelian insights with interactionist research clarifies that virtue involves both responsiveness to situational demands and consistent moral motivation. Two individuals may encounter identical external pressures but behave differently due to significant internal differences in how their reason, desire, and emotion are integrated within their character.

Therefore, trait-based views might note stable dispositions but miss the quality of motive and moral reasoning. Behavior-based views might see outward acts but miss the internal conflict (e.g., continent vs. virtuous). Situation-based views might see how context influences behavior but miss the moral agency that navigates and interprets contexts and acts in spite of or in response to situational characteristics.

## 4. A Functionalist Framework for Assessing Character Virtues

### 4.1. Moving Beyond Traits, Behaviors and Situations

To address the limitations of purely trait-based, behavior-based, and situation-based views, we can draw on functionalist perspectives that emphasize the role or purpose that beliefs and attitudes serve. In functionalism, mental representations, be they cognitive schemata, beliefs, or motivations, emerge and persist because they fulfill particular goals (Cummins, 1975). Likewise, personality psychologists have noted that a functionalist perspective can be taken to explain behavioral tendencies in terms of these constructs (Wood et al., 2015).

It is precisely these desired ends that, in part, differentiate the various character types from each other beyond the behavioral tendencies themselves. Applying this lens to moral character reveals new insights into why individuals who exhibit similar outward behaviors can nevertheless fall into very different Aristotelian character categories. Instead of merely noting that someone behaves compassionately because they engage in relevant behavior across salient situations, functionalism inquires: What function do those behaviors serve within the person’s overall moral aim?

The intertwining of reason, purpose, and action forms the core of motivated, rational activity, guiding not just what a person does, but why they deem an action appropriate or good (Korsgaard, 2008). By focusing on functions, we can ask what specific purposes or aims are served by an individual’s behavioral tendencies and how adaptive they are to their current circumstances. In turn, this perspective allows for a deeper analysis of lower-order, within-person constructs that can explain behavioral tendencies. These broadly include evaluations of one’s abilities to engage in certain behaviors, evaluations of how likely doing so will lead to different outcomes, and how desirable those outcomes are (Wood et al., 2015). For example, when it comes to how valued certain end states are, different people may prioritize self-consistency, maximize short-term pleasure, reduce conflict, or align with ethical ideals. In this light, moral beliefs both reflect and shape the “ends” a person pursues, clarifying why two apparently “honest” individuals might differ in their core motivations: one’s honesty may stem from a sincere commitment to a value internal to honesty as a virtue (e.g., truth), whereas the other’s might arise from a fear of punishment or disapproval. That is, to possess the virtue (e.g., of honesty), one must at least partially be intrinsically motivated by epistemic goods such as truth (Baehr, 2020). Although their actions look alike, the contrast in underlying motives reveals how one person acts out of virtuous alignment while the other merely complies to avoid negative outcomes.

Functionalist theories can examine how moral beliefs and attitudes help maintain the person’s moral system. These beliefs interact with intentions, motivations, and emotional responses, shaping how we perceive moral dilemmas and decide to act. From this perspective, beliefs can be viewed as components that either integrate smoothly or conflict with one another, thus shaping overall character and designating one’s character type. “Functionality indicators” (Wood et al., 2015) make that interaction tractable: they guide how we interpret situations, what we value, and how we decide to act. When beliefs are well-integrated, they support each other and encourage consistent moral action. For example, these functionality indicators (valuation, efficacy, expectancy) let us infer (a) the outcomes the system is tacitly organized to secure (function), and (b) the reasons the agent asserts (intention). A virtuous person’s beliefs align harmoniously, making moral choices feel natural. For instance, believing “honesty is crucial” and “lying damages trust” both reinforce a commitment to telling the truth. While the coherence of beliefs and motivations is a relevant indicator of psychological organization, it is important to clarify that coherence alone does not entail moral virtue. Indeed, rigid, overly simplistic, or ideologically extreme belief systems might be highly coherent but deeply immoral or vicious. For example, if someone’s dominant beliefs consistently endorse harmful actions (and no competing beliefs restrain them), that person can develop a vicious character. Thus, coherence is a descriptive rather than normative indicator.

When beliefs about how desirable certain end states are in conflict, for instance, truth vs. reputation, the overall moral system becomes strained. Consider a person expressing remorse and regret for killing another when on trial but not owning the act—without recognizing it as their own wrong and without intention to change their ways (Battaly, 2025). Procrastination, inaction, or moral back-and-forth may result as the person struggles to reconcile these incompatible components in a given situation. The way that these within-person representations interact and how well they cohere or contradict influences a person’s character type: beastly, vicious, incontinent, continent, or virtuous.

Thus, studying a person’s character from a functionalist perspective would mean examining the specific roles these functionality indicators play, how they fit together, and the ways in which the motivation that follows may energize or suppress certain behaviors. Beyond explaining and predicting behavioral patterns though, taking a functionalist perspective and assessing functionality (e.g., valuations or how desirable certain outcomes are) is arguably part of a theory-based approach to virtue assessment in its own right that has been neglected.

### 4.2. Functionalism and the Five Aristotelian Character Types

Let us look at the five character types again to functionally analyze each character type by using courage as the virtue in a train station emergency situation where a commuter is attacked on a crowded platform.

**Beastly.** A bystander rushes in to join the assault, driven purely by a surge of violent thrill.

*Functional Analysis*: Hedonic valuation of raw excitement; moral cognition is absent.

**Vicious.** Another onlooker films the beating, eager to post a viral clip and revel in the aggressor’s power. The victim’s suffering is entertaining because it promises online admiration.

*Functional Analysis:* Status-seeking valuation (likes, notoriety) that reframes harm as worthwhile.

**Incontinent.** A commuter knows he ought to help but freezes, overwhelmed by fear of injury. His endorsed moral end (“protect the victim”) is eclipsed by the impulse to avoid pain.

*Functional Analysis*: Expectancy of personal harm outweighs moral valuation.

**Continent.** Despite shaking, another commuter yanks the emergency lever and calls for help, forcing herself to act against strong self-preservation motives.

*Functional Analysis*: Self-regulatory efficacy is used to override conflicting valuations.

**Virtuous.** Another commuter steps between attacker and victim; intervening feels natural, guided by an ingrained commitment to safeguarding the vulnerable.

*Functional Analysis*: Fully integrated valuation + expectancy + efficacy—no inner conflict.

Thus, the identical outward behavior, which here is to intervene or not intervene, acquires its moral meaning from the function it serves within each agent’s overall moral aim.

## 5. Functionalist Utility in Character Assessment

A functionalist approach to assessing character virtues goes beyond static portraits of individuals or discrete behaviors. Functionalism (Wood et al., 2015; Wood et al., 2020) offers a perspective on moral dispositions and actions as goal-oriented strategies, meaning that behaviors become a means to an end. By examining the outcomes that people expect or the benefits they perceive, we can gain insights into the reasons behind ethical or unethical conduct (Ajzen, 1991; Carver & Scheier, 2001). The research by Williams et al. (2024) extended this perspective by demonstrating that virtues function within a broader framework of moral motivation. They emphasize that virtues are not merely fixed traits but develop dynamically in response to self-transcendent motivations. Their model explains how virtues emerge as goal-directed behaviors that align with a person’s broader purpose, particularly when shaped by self-transcendent values such as the desire to contribute to the well-being of others or to serve a greater good. Integrating a functionalist approach allows for a better understanding of virtues as adaptive, context-sensitive strategies that support moral development, rather than as static personality characteristics.

Viewed in this light, a behavior’s intended effect can be understood as its driving cause. This helps uncover the deeper “why” of moral choices. If someone routinely lies, functionalism asks which ends those lies serve: protecting the ego, protecting others, gaining status, avoiding discomfort, or even reflecting cynicism about moral norms (Wood et al., 2015). Consequently, the moral significance of an action depends in part on how it integrates with the individual’s broader system of beliefs, desires, and reasoning. Moreover, a functionalist perspective is consistent with the notion that virtue and its development involves pursuing those goals in the appropriate or right way (McManus et al., 2024; Ratchford et al., 2024) and perhaps choosing an action that is neither excessive nor deficient for the circumstances in which they are enacted (Ng & Tay, 2020; Ratchford et al., 2024). Thus, the functionalist perspective highlights not only the aim of an action, but also the perceived capacity and effectiveness in bringing about the intended effect.

In essence, functionalism aligns with Aristotle’s view that virtue is not just about what we do, but about *why* and *how* we do it. By exploring the goals embedded in moral cognition, this approach helps researchers and practitioners probe beneath the surface of actions. It is suitable for analyzing moral development, showing how one’s strategies for living both shape and are shaped by the ends one values (Wood et al., 2020). Such a perspective can guide efforts to measure and foster virtue by bridging philosophical concepts and empirical research on character.

## 6. Next Steps: Operationalizing a Functionalist Approach to Virtue Measurement

A major challenge in virtue assessment is the need for a systematic approach that captures both the external behavior and internal motivation. As Fowers (2014) argues, existing virtue assessments have struggled with construct validity, situational influences, and self-report biases (Ahrens & Cloutier, 2019; Fowers, 2014). To move beyond these limitations, researchers should develop a functionalist framework for assessing character virtues, grounded in the way that virtues operate within an individual’s moral reasoning, desires, and actions.

Existing psychological models such as the theory of planned behavior (TPB) and functional indicators (FIs) provide useful reference points for how a functionalist framework could be structured. TPB, for example, predicts behavior based on behavioral beliefs (anticipated outcomes), subjective norms (social pressures), and perceived behavioral control (self-efficacy) (Ajzen, 1991). Meanwhile, FIs use factors such as ability/efficacy (confidence in performing an action), expectancy (anticipated effects of the action), and valuation (importance placed on outcomes) (Wood et al., 2015). Researchers should use these frameworks as inspiration to develop a functionalist approach tailored to character virtues.

To build a functional assessment of character virtues, researchers should consider:

1. Defining Virtue-Specific Indicators—Virtues involve an alignment of moral cognition, affect, and desire or motivation. Measures should assess how an individual integrates moral beliefs and motivations across different situations rather than just their frequency or average level of enactment of moral behavior across situations.
2. Integrating Multi-Method Approaches—As Fowers (2014) suggests, virtue measurement should combine self-report scales, behavioral observations, and experience sampling to capture how moral character functions in real-life contexts. Self-report measures should be developed that assess not just the behavioral frequency, but also the perceived efficacy, expected moral outcomes, and desirability of outcomes. Behavioral experiments can test moral decision-making in real-world dilemmas, distinguishing between individuals acting effortlessly (virtuous) versus those struggling with internal conflict (continent/incontinent). Experience sampling can capture the daily moral choices to measure behavior and their respective motivations and aims over time and in situ.
3. Focusing on Moral Development—A functionalist perspective arguably allows for a more nuanced account of virtue development over time compared with treating and measuring them as static traits. This aligns with Aristotle’s emphasis on habituation and practical wisdom (phronesis) in moral character formation across the character types.
4. Clarifying Normative Anchors—Future studies should state whose standards ground their virtue judgments. Three viable positions are: participant-as-judge—the actor weighs the moral meaning of her own behavior, well-suited to within-person coherence research. Observer-as-judge—peers or community members supply locally shared standards (e.g., cultural conceptions of generosity). Researcher-as-judge—investigators apply an explicit philosophical framework (here, neo-Aristotelian) to behavioral and motivational evidence. Making these anchors explicit prevents the common slippage in which descriptive indicators are mistaken for virtue itself. Fowers et al. (2021) showed that virtue science must either disengage, that is remain descriptively neutral, or “bite the bullet” by declaring its evaluative standard.

## 7. Conclusions

In summary, this paper has advanced a novel perspective on virtue by integrating Aristotelian ethics with a functionalist framework. By looking at the importance of internal motivations and the interplay between beliefs, desires, and emotions, we have shown that genuine virtue extends beyond outward behavior to encompass a well-integrated moral character. Although traditional trait-, behavior-, and situation-based approaches offer valuable insights, they often fall short in distinguishing between superficially similar actions that differ in their underlying aims or purpose, which is a key feature of virtues. The functionalist framework, as argued, provides an approach that aligns with both classical philosophical insights and contemporary empirical research.

Nonetheless, potential criticisms merit further consideration. One might argue that reducing moral behavior to functional roles risks oversimplifying the complexity of human motivation. However, not accounting for the intentional component underlying virtue-relevant behavior as in the current approaches seems to risk not accounting for a constitutive feature of the virtues construct type. Additionally, the operationalization of such a framework in empirical research poses challenges, particularly in capturing any conflicting goals and internal motivations of moral reasoning and mental states more broadly, although methods that mentally situate respondents in specific situations prior to assessment seem to help in reducing certain biases (Brienza et al., 2018). Future research should therefore focus on developing innovative measurement tools and methodologies that can adequately reflect this complexity. Moreover, cross-disciplinary collaborations between philosophers and psychologists will be essential in refining these empirical models and ensuring that they remain faithful to the theoretical foundations of virtue ethics. By addressing these challenges and pursuing further empirical validation, this framework has the potential not only to deepen our theoretical understanding of virtue, but also to inform practical interventions aimed at fostering moral development in various social contexts.
